# Supplementary material for: CryoET of β-amyloid and tau within postmortem Alzheimer’s disease brain
Source: Nature. 2024 Jul 10;631(8022):913–9. doi: 10.1038/s41586-024-07680-x (PMC11269202; doi:10.1038/s41586-024-07680-x)
Supplement: Supplementary file 1 — Source data showing raw, uncropped immunoblots related to Fig. 1b and Extended Data Fig. 1b. [file 41586_2024_7680_MOESM1_ESM.pdf]

nature portfolio

https://doi.org/10.1038/s41586-024-07680-x

Supplementary information

CryoET of β-amyloid and tau within postmortem Alzheimer's disease brain

In the format provided by the authors and unedited

Related to Fig. 1b

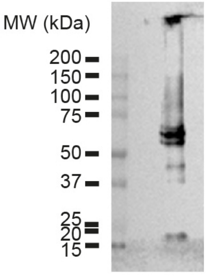

IB: Tau 46

Related to ED Fig. 1b

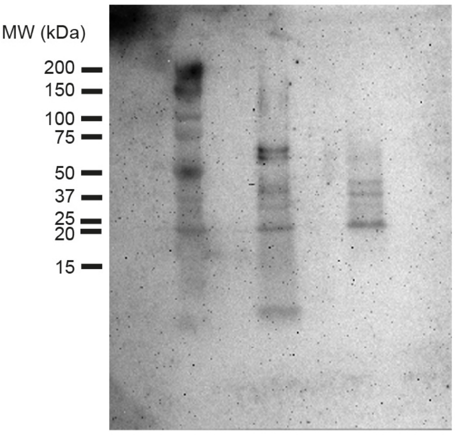

IB: AT8

Related to ED Fig. 1b

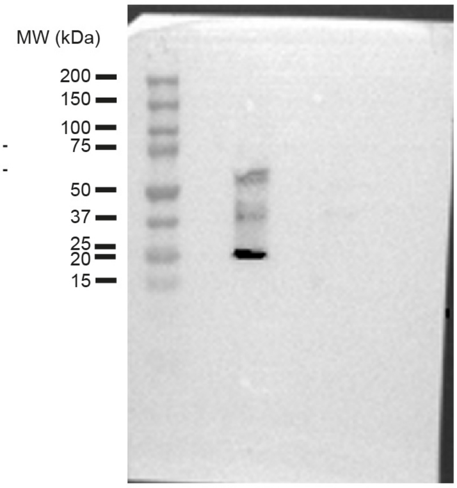

Tau 3R

Related to ED Fig. 1b

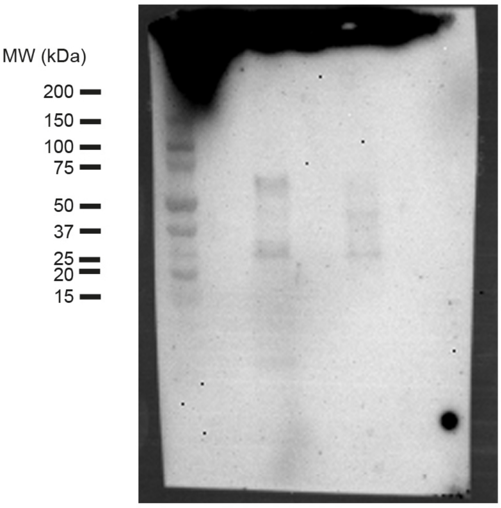

Tau 4R

Related to ED Fig. 1b

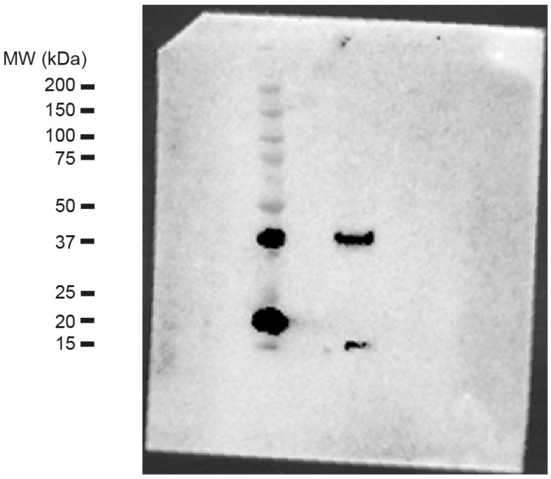

TMEM106B
