# Supplementary material for: CryoET of β-amyloid and tau within postmortem Alzheimer’s disease brain
Source: Nature. 2024 Jul 10;631(8022):913–9. doi: 10.1038/s41586-024-07680-x (PMC11269202; doi:10.1038/s41586-024-07680-x)
Supplement: Supplementary file 2 — Reporting Summary [file 41586_2024_7680_MOESM2_ESM.pdf]

Corresponding author(s): René Frank

Last updated by author(s): May 21, 2024

Reporting Summary

Nature Portfolio wishes to improve the reproducibility of the work that we publish. This form provides structure for consistency and transparency in reporting. For further information on Nature Portfolio policies, see our Editorial Policies and the Editorial Policy Checklist.

Statistics

For all statistical analyses, confirm that the following items are present in the figure legend, table legend, main text, or Methods section.

n/a	Confirmed	
☐	☒	The exact sample size (n) for each experimental group/condition, given as a discrete number and unit of measurement
☐	☒	A statement on whether measurements were taken from distinct samples or whether the same sample was measured repeatedly
☒	☐	The statistical test(s) used AND whether they are one- or two-sided<br>*Only common tests should be described solely by name; describe more complex techniques in the Methods section.*
☒	☐	A description of all covariates tested
☒	☐	A description of any assumptions or corrections, such as tests of normality and adjustment for multiple comparisons
☐	☒	A full description of the statistical parameters including central tendency (e.g. means) or other basic estimates (e.g. regression coefficient) AND variation (e.g. standard deviation) or associated estimates of uncertainty (e.g. confidence intervals)
☒	☐	For null hypothesis testing, the test statistic (e.g. F, t, r) with confidence intervals, effect sizes, degrees of freedom and P value noted<br>*Give P values as exact values whenever suitable.*
☒	☐	For Bayesian analysis, information on the choice of priors and Markov chain Monte Carlo settings
☒	☐	For hierarchical and complex designs, identification of the appropriate level for tests and full reporting of outcomes
☒	☐	Estimates of effect sizes (e.g. Cohen's d, Pearson's r), indicating how they were calculated

Our web collection on statistics for biologists contains articles on many of the points above.

Software and code

Policy information about availability of computer code

Data collection	CryoET: Tomo v5.15 (Thermo Fisher) ; cryoEM: EPU v3 (Thermo Fisher)
Data analysis	CryoET and cryoCLEM data processing:<br>MotionCorr2 v1.2.1, IMOD v4.12.35, PEET v1.17.0a, Warp/M v1.10b, RELION v4.0, em_placement v1.2.2, Dynamo v1.1.532, AreTomo v1.3.0,EMAN v2.99, Isonet v0.2, ChimeraX v1.5, Phenix v1.2.1, Zeiss ZEN Blue v3.6, Matlab R2019a, ImageJ v2.0.0-rc-49/1.51d, ArtiaX v0.4.7.<br>CryoEM data processing:<br>MotionCorr2 v1.2.1, RELION v4.0, crYOLO v1.9.6, Coot v0.8.9.2, Phenix v1.17.1, CTFFIND v1.14, Molprobity v4.5.2.

For manuscripts utilizing custom algorithms or software that are central to the research but not yet described in published literature, software must be made available to editors and reviewers. We strongly encourage code deposition in a community repository (e.g. GitHub). See the Nature Portfolio guidelines for submitting code & software for further information.

Data

Policy information about availability of data

All manuscripts must include a data availability statement. This statement should provide the following information, where applicable:

- Accession codes, unique identifiers, or web links for publicly available datasets
- A description of any restrictions on data availability
- For clinical datasets or third party data, please ensure that the statement adheres to our policy

A data availability statement is included. Subtomogram average maps have been deposited in the Electron Microscopy Data Bank (EMDB) under accession codes EMD-50148 (CS1, Extended Data Fig. 9c), EMD-50152 (CS2, Fig. 3g), EMD-50153 (CS3, Extended Data Fig 9b), EMD-50155 (CS4, Extended Data Fig 9d), EMD-50156 (CS5, Extended Data Fig 9a), EMD-50157 (CS6, Extended Data Fig 9e), EMD-50159 (CS7, Extended Data Fig 9f), EMD-50160 (LOL1 PHF, Fig. 4h), EMD-50161 (LOL1 SF, Fig 4h), EMD-50162 (LOL2 SF, Fig. 4j). The cryoEM map of sarkosyl-extracted tau PHF from post-mortem AD donor has been deposited to the EMDB with the accession code EMD-18990.
Dose-fractionated movie frames and tomograms associated with CS1-CS7 and LOL1-LOL2 subtomogram average maps have been deposited in the Electron Microscopy Public Image Archive (EMPIAR) under accession code EMPIAR-12082. Tomographic datasets of post-mortem AD brain tissue, non-demented control and AppNL-G-F knockin mice have been deposited under accession codesEMPIAR-12091, EMPIAR-12088 and EMPIAR-12092, respectively.

Research involving human participants, their data, or biological material

Policy information about studies with human participants or human data. See also policy information about sex, gender (identity/presentation), and sexual orientation and race, ethnicity and racism.

Reporting on sex and gender	Post-mortem Alzheimer's disease donor brain tissue: Sex: Female. Age at death: 70 years.<br>Post-mortem non-demented control donor brain tissue: Sex: Male. Age at death: 90 years.
Reporting on race, ethnicity, or other socially relevant groupings	This study did not include socially constructed or socially relevant variables.
Population characteristics	Between 70 and 90 year's old. Neuropathological diagnosis of Alzheimer's disease.
Recruitment	Samples were selected on the basis of neuropathological examination and brain tissue availability, which are unlikely to have impacted the results.
Ethics oversight	This study was performed at Netherlands Brain Bank, Amsterdam University Medical Centres (location VUmc), and the University of Leeds. This study was approved by both VUmc and the University of Leeds Research Ethics Committee. Informed consent was obtained from the patients' next of kin.

Note that full information on the approval of the study protocol must also be provided in the manuscript.

Field-specific reporting

Please select the one below that is the best fit for your research. If you are not sure, read the appropriate sections before making your selection.

☒ Life sciences ☐ Behavioural & social sciences ☐ Ecological, evolutionary & environmental sciences

For a reference copy of the document with all sections, see nature.com/documents/nr-reporting-summary-flat.pdf

Life sciences study design

All studies must disclose on these points even when the disclosure is negative.

Sample size	No sample size calculation was performed. Since population-based variation was not investigated in this study, a single post-mortem AD and non-demented control donor brain sample (mit-temporal gyrus) was used for cryoET. Cingulate cortex from the same donor was used for cryEM of sarkosyl-insoluble tau. A single 10 months-old, male App^NL-G-F/NL-G-F (Saito et al., 20214) mouse was used to confirm the effect of post-mortem interval.
Data exclusions	No tomograms were excluded from any of the datasets. For helical averaging of cryoET and cryoEM data, pre-established common image classification procedures (S.H.W. Scheres, J. Struc. Biol. 180: 519-530, (2012)) were employed to select particle images with the highest resolution content in the cryo-EM reconstruction process. Details of the number of selected images are given in Extended Data Table 5 and 8.
Replication	All attempts at replication were successful. At least three independent repeats per experiment where representative data is shown.
Randomization	Not applicable applicable to this study. Samples were allocated to experimental group on the basis of neuropathological examination. For subtomogram averaging, subvolumes were divided into two random halves for gold standard Fourier shell correlation estimates of resolution. The same is true of for the averaging of cryoEM data, where the particles were randomly divided into half sets during refinement in RELION.

Blinding

Identification of the molecular and organelle constituents of tomograms was performed blind by two independent curators. For all other experiments, investigators were not blinded to allocation during experiments and outcome assessment. The perceived risk of detection/performance bias was deemed negligible.

Reporting for specific materials, systems and methods

We require information from authors about some types of materials, experimental systems and methods used in many studies. Here, indicate whether each material, system or method listed is relevant to your study. If you are not sure if a list item applies to your research, read the appropriate section before selecting a response.

Materials & experimental systems

n/a	Involved in the study
☐	☒ Antibodies
☒	☐ Eukaryotic cell lines
☒	☐ Palaeontology and archaeology
☐	☒ Animals and other organisms
☒	☐ Clinical data
☒	☐ Dual use research of concern
☒	☐ Plants

Methods

n/a	Involved in the study
☒	☐ ChIP-seq
☒	☐ Flow cytometry
☒	☐ MRI-based neuroimaging

Antibodies

Antibodies used

Immunohistochemistry primary antibodies: 1:800 anti-pTauSer202/Thr205 clone AT8 (MN1020, ThermoFisher Scientific, Waltham, MA, USA), 1:1000 anti-amyloid beta clone 4G8 (#800710, Biolegend, San Diego CA, USA), 1:6400 anti-pTau-Thr217 (#44-744, ThermoFisher Scientific), 1:1000 anti-P62-lck (#610833, BD Biosciences, CA), 1:500 anti-alpha-synuclein (phospho-S129, clone EP1536Y) (ab51253, Abcam, Cambridge, UK), 1:6000 anti-pTDP-43 Ser409/410 (TiP-PTD-M01, Cosmo Bio USA, CA, USA), 1:1000 anti-TMEM106B (C terminal, SAB2106773, Sigma-Aldrich).
Immunohistochemistry secondary antibodies: Envision mouse/rabbit HRP (K4065, DAKO, Glostrup, Denmark).
Immunoblot primary antibodies: 1:2000 anti-Tau clone Tau46 (aa 404-441, T9450, Merck), 1:1000 anti-pTauSer202/Thr205 clone AT8 (MN1020, ThermoFisher), 1:1000 anti-4-repeat Tau (aa 275-291, 05-804, Merck), 1:500 anti-3-repeat Tau (aa 267-316, 05-803, Merck), 1:1000 anti-C-terminal domain TMEM106B (SAB2106778, Merck).
Immunoblot secondary antibodies: 1:20000 anti-mouse-HRP (170-6516, Bio-rad), 1:20000 anti-rabbit-HRP (172-1019, Bio-rad).
Immunofluorescence primary antibodies: 1:750 anti-amyloid beta clone 6E10 (803001, Biolegend), 1:750 anti-amyloid beta clone 4G8 (800710, Biolegend), 1:750 anti-pTauSer202/Thr205 clone AT8 (MN1020, Thermofisher).
Immunofluorescence secondary antibodies: 1:1000 anti-mouse IgG2B AF-633 (A21126, Thermo Fisher), 1:1000 anti-mouse IgG1 AF-568 (A21124, Thermo Fisher).

Validation

Immunohistochemistry primary antibodies: Anti-pTauSer202/Thr205 clone AT8: Validated extensively in the literature (e.g. Braak and Braak, Acta Neuropathol 82:239 (1991)) for immunohistochemical detection in human tissues. Anti-amyloid beta clone 4G8: Validated extensively in the literature (e.g. Forny-Germano L, et al. J Neurosci. 34:13629 (2014)) for immunohistochemical detection in human tissues. Anti-pTau-Thr217: Validated by Hart de Ruyter F, et al. Acta Neuropathol 145(2):197-218 (2023) for immunohistochemical detection in human tissues. Anti-P62-lck: Validated extensively in the literature (e.g. Kovacs G, et al. Neuropathology and Applied Neurobiology. 39:166-78 (2013)) for immunohistochemical detection in human tissues. Anti-alpha-synuclein (phospho-S129): Validated extensively in the literature (e.g. Lashuel et al. NPJ Parkinson Dis, 8:136 (2023); Delic et al., J Comp Neurol. 526:1978 (2018)) for immunohistochemical detection in human tissues. Anti-pTDP-43 Ser409/410: Validated extensively on human tissues in the literature (e.g. Kovacs et al., Neuropathology and Applied Neurobiology. 39:166 (2013)) for immunohistochemical detection in human tissues. Anti-TMEM106B: Validated by Perneel J, et al., Acta Neuropathologica 145:285 (2022) for immunohistochemical detection in human tissues.
Immunohistochemistry secondary antibodies: Anti-mouse/rabbit HRP (DAKO, Glostrup, Denmark): Validated in many other studies (e.g. Hart de Ruyter et al., Acta Neuropathol 145:197 (2023); Boon et al., Acta Neuropathol 140:811 (2020).
Immunoblot primary antibodies: anti-Tau clone Tau46 : Validated by Mawal-Dewan et al., J. Biol. Chem. 269:30981 (1994) for detection of human tau. Anti-pTauSer202/Thr205 clone AT8: Validated against human pS202/pT205 tau in (Mercken et al., Acta Neuropathol. 84, 265-272 (1992)) for detection of human tau. Anti-4-repeat Tau: Validated by Ercan et al., Molecular Neurodegeneration 12:87 (2017) for detection of human tau. Anti-3-repeat Tau: Validated by Ercan et al., Molecular Neurodegeneration 12:87 (2017) for detection of human tau. Anti-C-terminal domain TMEM106B: Validated by Perneel J, et al., Acta Neuropathologica 145:285 (2022) for detection of human TMEM106B Anti-amyloid beta clone 6E10: Validated in earlier study using wild-type control (Leistner et al., Nat. Commun. 14:2833 (2023)) for detection of human beta-amyloid. Anti-amyloid beta clone 4G8: Validated in many earlier study (e.g. Poduslo et al., Biochem. 43:6064 (2004)) for detection of human beta-amyloid.
Immunoblot secondary antibodies (anti-mouse-HRP and anti-rabbit-HRP): Validated in manufacturer's (Bio-rad) website: https://www.bio-rad.com/en-uk/product/hrp-ap-conjugates?ID=cc14540b-25dd-4bbf-a17a-8c3c52116d9d): "Blotting-grade HRP conjugates (horseradish peroxidase) and AP conjugates (alkaline phosphatase) produce specific results, eliminating false positives in western blotting immunoassays: Double affinity-purified blotting-grade antibodies are isolated by affinity chromatography and further purified by cross-adsorption against an unrelated species to eliminate nonspecific immunoglobulins".
Immunofluorescence primary antibodies: Anti-pTauSer202/Thr205 clone AT8: Validated by Dehkordi et al. Nat. Aging 1:1107 (2021). Anti-amyloid beta clone 4G8: Validated in many earlier study (e.g. Poduslo et al., Biochem. 43:6064 (2004)).
Immunofluorescence secondary antibodies (anti-mouse IgG2B AF-633, anti-mouse IgG1 AF-568): Validated extensively in the literature (e.g. Leistner et al., Nat. Commun. 14:2833 (2023).

Animals and other research organisms

Policy information about studies involving animals; ARRIVE guidelines recommended for reporting animal research, and Sex and Gender in Research

Laboratory animals	10 month old App^NL-G-G/NL-G-F knockin mouse on a c57b/l6 background (Saito et al., Nat. Neurosci. 17:661 (20214). Housing conditions: 20–24°C , 12 hour day/night cycle, 45-65% relative humidity.
Wild animals	The study did not involve wild animals.
Reporting on sex	Only male animals were used in the study. Sex based analysis is not necessary because differences in the architecture of amyloid plaques is not expected to be significantly different in the male versus female App.
Field-collected samples	Study did not involve field-collected samples.
Ethics oversight	Oversight and approval was provided by the University of Leeds Animal Welfare and Ethics Review Board and licensed by the UK Government Home Office.

Note that full information on the approval of the study protocol must also be provided in the manuscript.

Plants

Seed stocks	This study did not include plants.
Novel plant genotypes	This study did not include plants.
Authentication	This study did not include plants.
