# Supplementary material for: CryoET of β-amyloid and tau within postmortem Alzheimer’s disease brain
Source: Nature. 2024 Jul 10;631(8022):913–9. doi: 10.1038/s41586-024-07680-x (PMC11269202; doi:10.1038/s41586-024-07680-x)
Supplement: Supplementary file 11 — Cryo-EM data collection and refinement statistics for the extracted AD fibril dataset. As the refined model was essentially identical to several previously published tau PHF fibril structures, including the starting template (PDB 5o3l), the final model from these data was not deposited to the PDB but is available on request. [file 41586_2024_7680_MOESM11_ESM.pdf]

	Ex vivo AD donor 12-054 (EMDB-18990)
**Data collection and processing**	
Magnification	96,000
Voltage (kV)	300
Detector	Falcon4
Pixel size (Å)	0.83
Electron exposure (e^{-}/Å^{2})	44
Exposure rate (e^{-}/pixel/s)	7.6
Nominal defocus range (μm)	-1.5 to -2.7
Movies collected	10,860
Initial particle images (no.)	321,041
Final particle images (no.)	40,180
Symmetry imposed	C1
Map resolution (Å)	3.0
FSC threshold	0.143
Map resolution range (Å)	2.8-4.5
Helical parameters	
Helical twist (°)	179.44
Helical rise (Å)	2.405
Crossover (nm)	~80
**Refinement***	
Initial model used (PDB code)	5O3L
Map sharpening *B* factor (Å^{2})	-57
Model resolution (Å)	2.9
FSC threshold	0.5
Model to map correlation	0.89
Cα R.M.S.D to initial model (Å)	0.63 (70 atoms)
